# Simulative learning in the room of horror – a method to enhance patient safety in undergraduate nursing education

**DOI:** 10.3205/zma001743

**Published:** 2025-04-15

**Authors:** Vivian Hauff, Laura Homann, Antje Tannen

**Affiliations:** 1 Charité – Universitätsmedizin Berlin, corporate member of Freie Universität Berlin and Humboldt-Universität zu Berlin, Institute of Clinical Nursing Science, Berlin, Germany

**Keywords:** nursing education, simulative learning, patient safety

## Abstract

**Objective:**

High expectations are placed on healthcare systems concerning safety and health restoration. Simultaneously, healthcare involves risks and potential hazards that may lead to adverse events for patients and healthcare professionals alike. To raise awareness of these risks, it is essential to incorporate the topic of patient safety into healthcare education. The *room of horror*, a form of simulated learning, represents an effective teaching and learning approach for this purpose.

**Methods:**

At the end of their first semester, undergraduate nursing students participated in a* room of horror* exercise designed following the Swiss manual for interactive learning. The task involved identifying 13 errors relevant to patient safety within the room. Subsequently, the students provided written evaluations of this teaching format.

**Results:**

Participants successfully identified twelve out of the thirteen safety-critical errors. All students perceived the simulation as educational and pertinent to professional practice. Heightened risk awareness and relevance to the professional context were particularly highlighted as positive outcomes.

**Conclusion:**

The *room of horror* provides a practical simulation training environment where students can develop observational skills, critical thinking, and situational awareness regarding patient safety risks early in their clinical education.

## Zusammenfassung

**Zielsetzung:**

An die Gesundheitsversorgung werden hohe Erwartungen in Bezug auf Sicherheit und Wiederherstellung der Gesundheit gestellt. Gleichzeitig birgt die Versorgung auch Risiken und potenzielle Gefahrenquellen, die zu unerwünschten Ereignissen sowohl bei Patient:innen als auch Gesundheitsfachkräften führen können. Um für diese Risiken zu sensibilisieren, ist es notwendig, das Thema Patientensicherheit schon in der Ausbildung der Gesundheitsprofessionen zu implementieren. Eine dafür geeignete Lehr- und Lernmethode stellt der *Room of Horror*, als eine Form des simulativen Lernens, dar.

**Methodik:**

Studierende des ersten Fachsemesters des Bachelorstudiengangs Pflege durchliefen am Semesterende einen *Room of Horror*, welcher in Anlehnung an das Schweizer Manual zum interaktiven Lernen konzipiert wurde. Es galt, 13 Fehler in diesem Raum zu identifizieren, die für die Patientensicherheit als relevant erachtet wurden. Im Anschluss fand eine schriftliche Evaluation dieses Lehrformats statt.

**Ergebnisse:**

Die Teilnehmenden waren in der Lage, zwölf der 13 Fehlerquellen zu identifizieren. Die Simulation wurde von allen Pflege-Studierenden als lehrreich und relevant für den beruflichen Alltag empfunden. Die Schärfung des Risikobewusstseins und die hohe Relevanz für den beruflichen Alltag sind Aspekte, die von den Teilnehmenden besonders positiv hervorgehoben wurden.

**Schlussfolgerung:**

Der *Room of Horror* stellt ein alltagsbezogenes Simulationstraining dar, in welchem die Studierenden frühzeitig ihre Beobachtungsfähigkeiten, kritisches Denken und Situationsbewusstsein für patientensicherheitsrelevante Gefährdungspotenziale im Klinikalltag trainieren können.

## 1. Introduction

Approximately ten percent of hospitalized patients worldwide experience adverse events during their stay, leading to harm [1]. Hospital care involves complex situations requiring extensive knowledge and skills from healthcare professionals [2]. Nurses play a critical role in patient safety, such as in early risk detection and safely executing nursing interventions [3], [4]. Addressing these challenges necessitates a fundamental engagement throughout nursing education, emphasizing both knowledge acquisition and the development of risk awareness [2], [3]. Simulative learning using the *room of horror* (RoH) is a suitable method to cultivate sensitivity to patient safety and train risk awareness among students [5], [6]. Positive effects of this teaching and learning method have been widely demonstrated, highlighting its role in developing safety-related skills [6]. This project aimed to evaluate the method and gain initial insights into its feasibility and acceptance among students.

## 2. Project description

**Figure 1:**
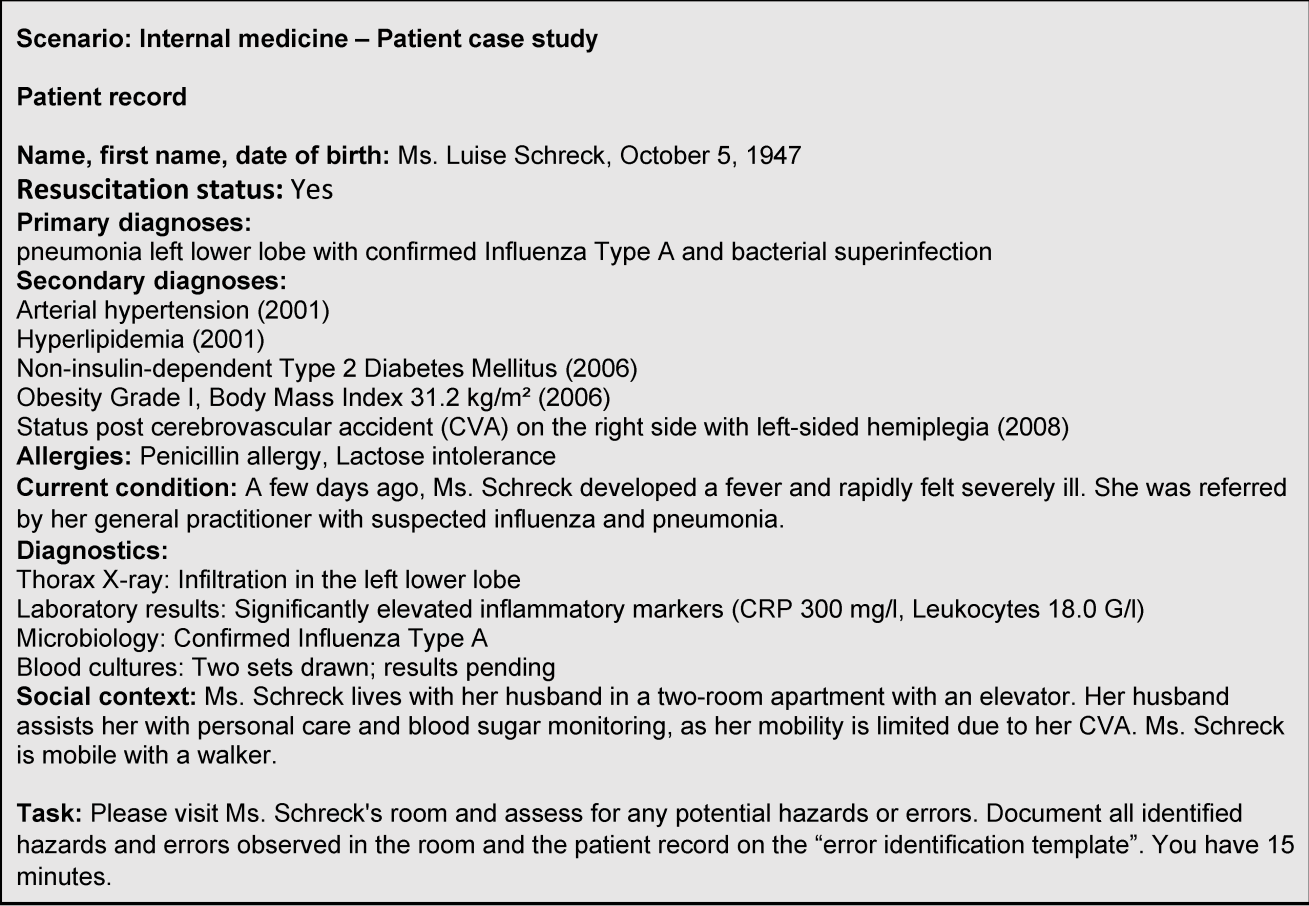
Case study example (extracted from [7])

Based on the WHO model curriculum [3], first-semester nursing students received comprehensive instruction on patient safety. Topics ranged from clarifying key terms and concepts, illustrating the roles of patients and nurses, to identifying risks associated with healthcare practices. At the end of the 12-week seminar series, students applied this knowledge by identifying errors in two identical simulated patient rooms equipped with a nursing simulation mannequin. No clinical placements had been conducted prior to this exercise. A total of N=42 students participated in the RoH over three consecutive days. The 90-minute simulation included theoretical preparation, pre-briefing, and debriefing. The pre-briefing involved a 20-minute case presentation (see figure 1 (Fig. 1)) and explanation of the RoH process. During the allotted time (15 minutes), two small groups of five to six students each simultaneously searched for errors. No differentiated role assignments were made. The rooms contained 13 safety-relevant errors based on the Swiss Patient Safety Foundation’s Manual [7] (see table 1 (Tab. 1)). Students documented identified errors on a recording sheet. Afterward, a joint analysis of the simulation was conducted, followed by a debriefing to discuss emotions and experiences. Participants then provided feedback via a brief paper-pencil survey using a four-point Likert scale. Quantitative data were descriptively analyzed (SPSS 29), while qualitative free-text responses were summarized.

## 3. Results

**Figure 2:**
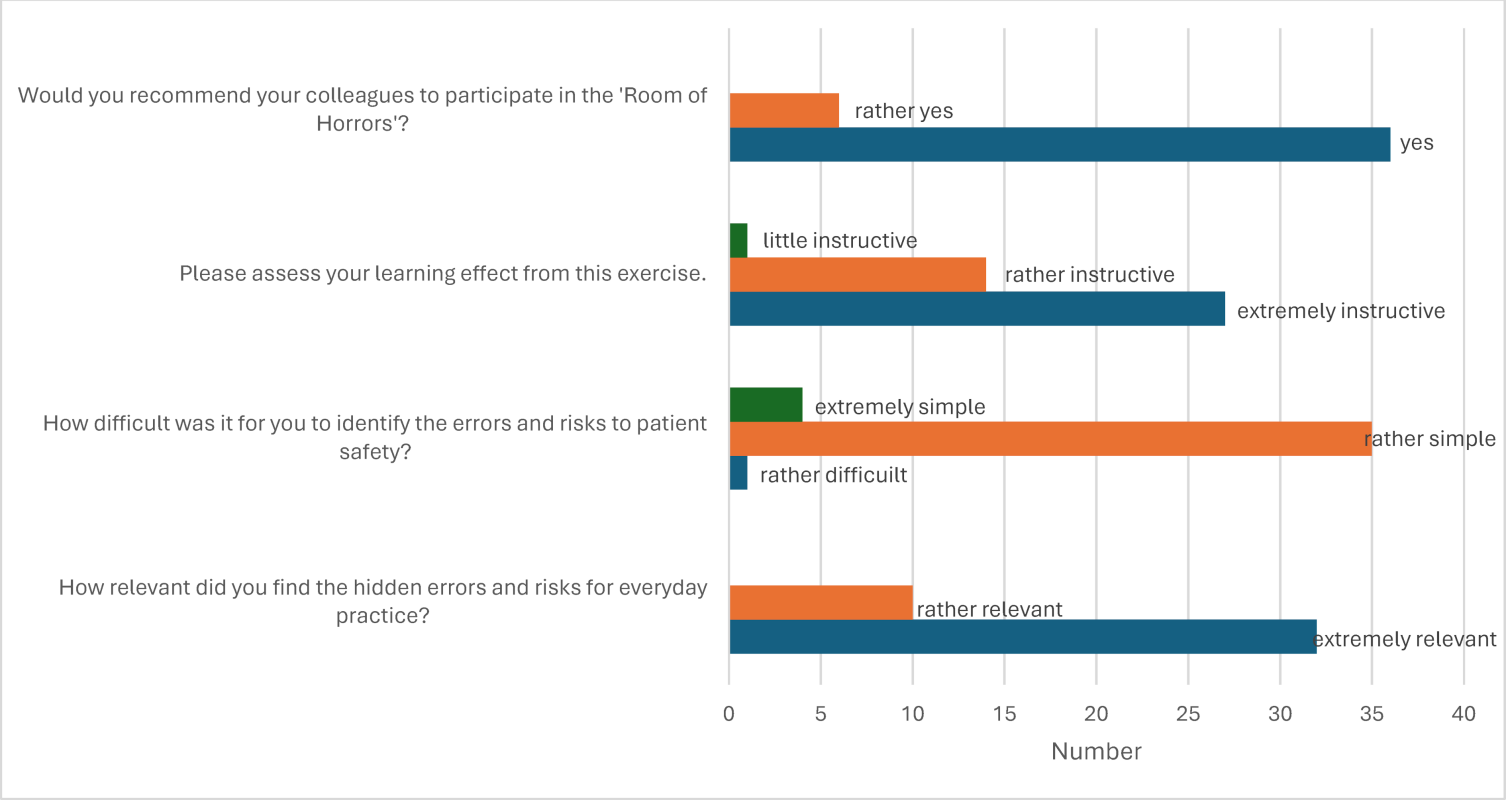
Evaluation results

Teams identified more errors in the simulation rooms than originally placed. However, a differentiated detection rate was not recorded. Errors such as incorrect patient positioning, an open urinary catheter, and empty hand sanitizer were recognized by all participants. Missing isolation precautions for documented influenza infection were overlooked by nearly all groups. Evaluation forms indicated that all students found the simulation relevant and educational for professional practice and would recommend participation to peers (see figure 2 (Fig. 2)). Open-ended questions asked about significant experiences, suggestions, and ideas for future RoH designs. Students emphasized the high relevance of the format for professional practice and its effectiveness in raising awareness of risks in patient care. They also highlighted the importance of a constructive error culture as a learning opportunity. Additionally, students suggested incorporating RoH exercises throughout their studies to simulate increasingly complex situations. Realistic room design and smaller group sizes of fewer than four participants were recommended to optimize the RoH experience. Overall, students found the simulation engaging and enjoyable.

## 4. Discussion

This RoH implementation suggests it is an effective and feasible teaching and learning method for raising awareness of patient safety [7]. It should be noted that successful implementation relies on personnel, logistical, and structural resources, which could present potential barriers requiring preemptive resolution. These findings align with other studies on simulative learning using the RoH [6]. Similar positive sentiments and high detection rates were reported in this project [6], [8]. Given that apparent errors like improper positioning or open urinary catheters were frequently identified, future exercises could focus on less obvious errors, such as documentation discrepancies. This pilot project’s evaluation is limited due to the small number of participants. Generalizations are not possible without replication in other studies and populations and testing the method in varied settings. Nonetheless, even within this relatively small cohort, positive effects such as perceived learning success and enhanced risk awareness were evident.

## 5. Conclusion

Simulative learning in a RoH is a low-threshold and educational method for promoting patient safety awareness in professional practice. Implementation is feasible for both basic and continuing education for nursing professionals. Elements such as realistic room design and optimal group sizes are critical for effectively training critical thinking and situational awareness. A repetitive format aligned with evolving qualifications and potential interprofessional orientation for real-world relevance would be desirable for future RoH designs.

## Authors’ ORCIDs

- Vivian Hauff: [0009-0000-1754-1952]
- Antje Tannen: [0000-0003-0970-1818]

## Competing interests

The authors declare that they have no competing interests. 

## Figures and Tables

**Table 1:**
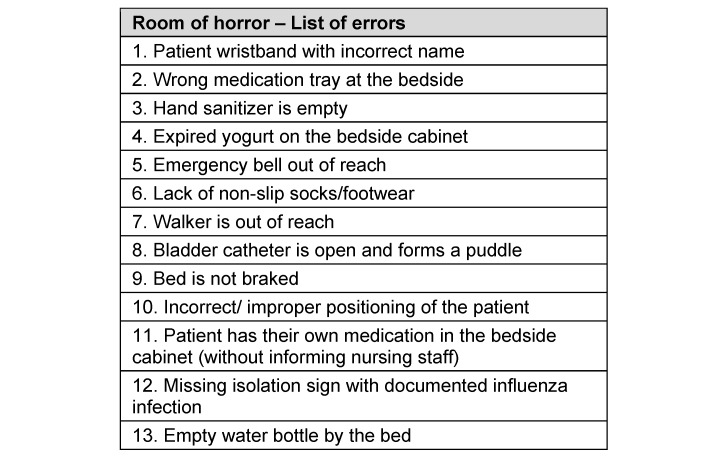
List of errors (extracted from [7] and adapted)
